# Supplementary material for: Microbiome Changes after Type 2 Diabetes Treatment: A Systematic Review
Source: Medicina (Kaunas). 2021 Oct 11;57(10):1084. doi: 10.3390/medicina57101084 (PMC8540512; doi:10.3390/medicina57101084)
Supplement: Supplementary file 1 [file medicina-57-01084-s001.zip › medicina-1366386-supplementary/S1_table.pdf]

Table S1. Decreased specific genera and species in *Firmicutes* phylum with corresponding clinical outcomes after any T2D treatment.

Genus	Species	RCT	Achieved outcome(s)
↓: Enterococcus	Faecalis	Su et al. [16]	↓ Lipid profile, inflammatory results
↓: Roseburia	Inulinivorans, Intestinalis, Hominis	Gu et al. (Acarbose arm) [17]	↓ Glycemic, lipid profile, anthropometric results
↓: Ruminococcus	Lactaris		
↓: Veillonella	Atypica		
↓: Eubacterium	Dolichum, Eligens, Ventriosum, Siraeum		
↓: Clostridium	Bolteae, Leptum, Spiroforme, Scindens		
↓: Holdemania	Filiformis		
↓: Pseudoflavonifractor	Capillosus		
↓: Anaerotruncus	Colihominis		
↓: Blautia	Hansenii		
↓: Ruminococcaceae	Bacterium		
↓: Lachnospiraceae	unclassified		
↓: Clostridium	–	Tong et al. (Prebiotic arm) [18]	↓ Glycemic, lipid profile, anthropometric results
↓: Clostridiales	–		
↓: Clostridium	Perfringens (E str. JGS1987), Perfringens (CPE str. F4969), Botulinum (C str. Eklund), Botulinum (E1 str. 'BoNT E Beluga'), Butyricum, Beijerinckii, unclassified	Wu et al. [19]	↓ Glycemic results
↓: Lactobacillus	Delbrueckii		
↓: Streptococcus	Thermophilus		
↓: Listeria	Seeligeri		
↓: Veillonella	unclassified, oral taxon		
↓: Clostridium	–	Shin et al. [29]	↓ Glycemic, inflammatory results, ↑ HR
↓: Oscilibacter	–		
↓: changes were present only at phylum level		Balfego et al. [30]	↓ Glycemic, anthropometric results
↓: Gemella	Sanguinis	Zhang et al. (Prebiotic arm) [31]	↓ Glycemic, lipid profile results
↓: Roseburia	Hominis, Inulinivorans, Intestinalis		
↓: Faecalibacterium	Prausnitzii		
↓: Coprococcus	Eutactus		
↓: Veillonella	Parvula		
↓: Butyrivibrio	Crossotus		
↓: Subdoligranulum	Variabile		
↓: Eubacterium	Siraeum, Eligens		
↓: Streptococcus	Sanguinis, Australis		
↓: Ruminococcus	Lactaris		
↓: Clostridium	Perfringens, Bartlettii		
↓: Ruminococcaceae	unclassified (D16)	Zhang et al. (Symbiotic arm) [31]	↓ Glycemic, lipid profile results
↓: Roseburia	Intestinalis, Inulinivorans, Hominis		
↓: Faecalibacterium	Prausnitzii		
↓: Coprococcus	Eutactus		
↓: Veillonella	Parvula, unclassified (sp. oral taxon 158)		
↓: Butyrvibrio	Crossotus		
↓: Subdoligranum	Variabile		
↓: Eubacterium	Siraeum, Eligens, Dolichum		
↓: Streptococcus	Sanguinis, Australis, Anginosus, Gordonii		
↓: Gemella	Sanguinis		
↓: Ruminococcus	Lactaris, Bromii		
↓: Clostridium	Perfringens, Bartlettii, unclassified (sp. L2-50), unclassified (sp. D5)		

↓ – decreased abundance of genus and / or species after applied treatment. "–" means that a certain parameter was not evaluated, achieved, or provided in a specific trial. HR – heart rate; RCT – randomized controlled trial.
