# Supplementary material for: Microbiome Changes after Type 2 Diabetes Treatment: A Systematic Review
Source: Medicina (Kaunas). 2021 Oct 11;57(10):1084. doi: 10.3390/medicina57101084 (PMC8540512; doi:10.3390/medicina57101084)
Supplement: Supplementary file 1 [file medicina-57-01084-s001.zip › medicina-1366386-supplementary/S2_table.pdf]

Table S2. Increased specific genera and species in *Firmicutes* phylum with corresponding clinical outcomes after any T2D treatment.

Genus	Species	RCT	Achieved outcome(s)
↑: Ruminococcus	Torques	Gu et al. (Acarbose arm) [17]	↓ Glycemic, lipid profile, anthropometric results
↑: Veillonella	Parvula		
↑: Megasphaera	Elsdenii		
↑: Acidaminococcus	Fermentans		
↑: Collinsella	Aerofaciens		
↑: Streptococcus	Salivarius, Vestibularis, Thermophilus, Infantis, Pneumoniae		
↑: Lactobacillus	Salivarius, Gasseri, Amylovorus, Oris, Crispatus, Rhamnosus		
↑: Megamonas	–	Tong et al. (Prebiotics arm) [18]	↓ Glycemic, lipid profile, anthropometric results
↑: Faecalibacterium	–		
↑: Blautia	–		
↑: Lachnospiraceae	–		
↑: Megamonas	–	Tong et al. (Metformin arm) [18]	↓ Glycemic, lipid profile, anthropometric results, ↑ dBP
↑: Blautia	–		
↑: Subdoligranulum	Variabile	Wu et al. [19]	↓ Glycemic results
↑: Lactobacillus	Ruminis, Vaginalis, Brevis, Sakei, Clariflavum		
↑: Eubacterium	Limosum		
↑: Megasphaera	Elsdenii		
↑: Clostridium	Botulinum (A3 str. Loch Maree), Botulinum (A3 str. Loch Maree) Bartlettii, Sticklandii, Hiranonis		
↑: Bacillus	Coahuilensis, Cereus		
↑: Streptococcus	Infantarius, Dysgalactiae		
↑: Lysteria	Grayi		
↑: Lactococcus	Garvieae		
↑: Granulicatella	Elegans		
↑: Veillonella	Dispar		
↑: Alkaliphilus	Metalliredigens, Oremlandii		
↑: Ruminococcus	unclassified		
↑: Dialister	–	Cortez et al. [20]	↓ Anthropometric results
↑: Roseburia	Intestinalis	Murphy et al. [21]	↓ Glycemic, anthropometric results
↑: Faecalibacterium	–	Lee et al. (RYGB arm) [22]	↓ Glycemic, anthropometric results
↑: Roseburia	–	Lee et al. (Control arm) [22]	↓ Glycemic, anthropometric results
↑: Lactobacillus	Reuteri	Mobini et al. [23]	↓ Glycemic results
↑: Lactobacillus	–	Firouzi et al. [24]	↓ Glycemic results
↑: Lactobacillus	Leptum, Gasseri, Casei, Reuteri	Sato et al. [25]	↓ Glycemic, lipid profile, inflammatory results
↑: Enterococcus	–		
↑: Clostridium	Coccoides		
↑: Lactobacillus	Reuteri	Hsieh et al. [26]	↓ Glycemic, lipid profile, inflammatory results, sBP
↑: Faecalibacterium	Prausnitzii	Medina-Vera et al. [27]	↓ Glycemic, lipid profile, inflammatory results, FFAs
↑: Megamonas	–	Shin et al. [29]	↓ Glycemic, inflammatory results; ↑ HR
↑: Mobilitalea	–		
↑: Acetivibrio	–		
↑: Lactobacillus	–		
↑: Lactobacillus	Rhamnosus, Ferementum, Plantarum, Casei, Gasseri, Crispatus, Salivarius	Zhang et al. (Probiotic arm) [31]	↓ Lipid profile results
↑: Holdemania	Filiformis		
↑: Dialister	Invisus		
↑: Clostridium	Bolteae		

S2 table (*continued*).

Genus	Species	RCT	Achieved outcome(s)
↑: Lactobacillus	Gasseri	Zhang et al. (Prebiotic arm) [31]	↓ Glycemic, lipid profile results
↑: Erysipelotrichaceae	unclassified		
↑: Lachnospiraceae	unclassified		
↑: Dorea	Longicatena		
↑: Anaerostipes	Caccae		
↑: Solobacterium	Moorei		
↑: Ruminococcus	Torques, Gnavus		
↑: Clostridium	Ramosum, Bolteae, Difficile		
↑: Lactobacillus	Rhamnosus, Ferementum, Plantarum, Casei, Gasseri, Crispatus, Salivarius	Zhang et al. (Symbiotic arm) [31]	↓ Glycemic, lipid profile results
↑: Erysipelotrichaceae	unclassified (5 2 54FAA), unclassified (3 1 53)		
↑: Anaerostipes	Caccae		
↑: Solobacterium	Moorei		
↑: Ruminococcus	Gnavus, Torques		
↑: Clostridium	Ramosum, Difficile, Bolteae, unclassified (sp. HGF2)		

↑ – increased abundance of genus and / or species after applied treatment. "–" means that a certain parameter was not evaluated, achieved, or provided in a specific trial. RCT – randomized controlled trial; dBP – diastolic blood pressure; FFAs – free fatty acid; HR – heart rate; RYGB - Roux-en-Y gastric bypass; sBP – systolic blood pressure.
