# Supplementary material for: Microbiome Changes after Type 2 Diabetes Treatment: A Systematic Review
Source: Medicina (Kaunas). 2021 Oct 11;57(10):1084. doi: 10.3390/medicina57101084 (PMC8540512; doi:10.3390/medicina57101084)
Supplement: Supplementary file 1 [file medicina-57-01084-s001.zip › medicina-1366386-supplementary/S3_table.pdf]

Table S3. Specific genera and species alterations in *Bacteroidetes* phylum with corresponding clinical outcomes after any T2D treatment

Genus	Species	RCT	Achieved outcome(s)
↓: Bacteroides	Clarus, Finegoldii, Intestinalis, Caccae, Xylanisolvens, Thetaiotaomicron, Stercoris, Uniformis, Plebeius, Dorei/Vulgatus	Gu et al. (Acarbose arm) [17]	↓ Glycemic, lipid profile, anthropometric results
↓: Alistipes	Shahii, Putredinis		
↓: Odoribacter	Splanchnicus		
↓: Capnocytophaga	–		
↓: Bacteroides	–	Tong et al. (Prebiotic arm) [18]	↓ Glycemic, lipid profile, anthropometric results
↓: Alistipes	–		
↓: Bacteroides	–	Tong et al. (Metformin arm) [18]	↓ Glycemic, lipid profile, anthropometric results, ↑ dBP
↓: Alistipes	–		
↓: changes were present only at phylum level		Cortez et al. (Control arm) [20]	–
↓: changes were present only at phylum level		Murphy et al. (RYGB arm) [21]	↓ Glycemic, anthropometric results
↓: Prevotella	Copri	Medina-Vera et al. [27]	↓ Glycemic, lipid profile, inflammatory results, FFAs
↓: Alloprevotella	–	Shin et al. [29]	↓ Glycemic, inflammatory results, ↑ HR
↓: Parabacteroides	Merdae	Zhang et al. (Prebiotic arm) [31]	↓ Glycemic, lipid profile results
↓: Prevotella	Copri		
↓: Alistipes	Shahii, Putredinis		
↓: Bacteroides	Caccae, Coprophilus, Pectinophilus		
↓: Paraprevotella	Xylaniphila	Zhang et al. (Symbiotic arm) [31]	↓ Glycemic, lipid profile results
↓: Parabacteroides	Merdae		
↓: Prevotella	Copri, Bivia		
↓: Alistipes	Shahii,		
↓: Bacteroides	Caccae, Coprophilus, Pectinophilus, Plebeius		
↑: Prevotella	Copri	Gu et al. (Acarbose arm) [17]	↓ Glycemic, lipid profile, anthropometric results
↑: Parabacteroides	Distasonis		
↑: Paraprevotella	–	Tong et al. (Prebiotic arm) [18]	↓ Glycemic, lipid profile, anthropometric results
↑: Capnocytophaga	Gingivalis	Wu et al. [19]	↓ Glycemic results
↑: Porphyromonas	Uenonis		
↑: Prevotella	Oris		
↑: Bacteroides	Clarus, Coprophilus		
↑: Bacteroides	–	Cortez et al. [20]	↓ Anthropometric results
↑: changes were present only at phylum level		Murphy et al. (SG arm) [21]	↓ Glycemic, anthropometric results
↑: Prevotella	–	Balfego et al. [30]	↓ Glycemic, anthropometric results
↑: Bacteroides	–		
↑: Parabacteroides	–	Zhang et al. (Prebiotic arm) [31]	↓ Glycemic, lipid profile results
↑: Capnocytophaga	Distasonis		
↑: Bacteroides	Dorei, Eggerthii, Fragilis, Ovatus, Vulgatus, Stercoris, Finegoldii, Clarus, Xylanisolvens		
↑: Bacteroides	Caccae	Zhang et al. (Probiotic arm) [31]	↓ Lipid profile results
↑: Odoribacter	Splanchnicus	Zhang et al. (Symbiotic arm) [31]	↓ Glycemic, lipid profile results
↑: Bacteroides	Dorei/Vulgatus, Dorei, Eggerthii, Fragillis, Ovatus, Stercoris, Finegoldii, Clarus, Thetaiotaomicron		

↓ – decreased abundance of genus and / or species after applied treatment. ↑ – increased abundance of genus and / or species after applied treatment. "–" means that a certain parameter was not evaluated, achieved, or provided in a specific trial. RCT – randomized controlled trial; dBP – diastolic blood pressure; FFAs – free fatty acid; HR – heart rate; RYGB - Roux-en-Y gastric bypass.
