# Supplementary material for: Microbiome Changes after Type 2 Diabetes Treatment: A Systematic Review
Source: Medicina (Kaunas). 2021 Oct 11;57(10):1084. doi: 10.3390/medicina57101084 (PMC8540512; doi:10.3390/medicina57101084)
Supplement: Supplementary file 1 [file medicina-57-01084-s001.zip › medicina-1366386-supplementary/S4_table.pdf]

Table S4. Specific genera and species alterations in *Actinobacteria* phylum with corresponding clinical outcomes after any T2D treatment

Genus	Species	RCT	Achieved outcome (s)
↓: Eggerthella,	Lenta	Gu et al. (Acarbose arm) [17]	↓ Glycemic, lipid profile, anthropometric results
↓: Bifidobacterium	–	Shin et al. [29]	↓ Glycemic, inflammatory results, ↑ HR
↓: Eggerthella	Lenta,	Zhang et al. (Symbiotic arm) [31]	↓ Glycemic, lipid profile results
↓: Bifidobacterium	Catenulatum, Adolescentis, Longum		
↓: Collinsella	Aerofaciens		
↓: Collinsella	Catenulatum, Adolescentis, Breve, Longum	Zhang et al. (Prebiotic arm) [31]	↓ Glycemic, lipid profile results
↓: Bifidobacterium	Aerofaciens		
↑: Bifidobacterium	Longum	Su et al. [16]	↓ Lipid profile, inflammatory results
↑: Bifidobacterium	Breve, Dentium, Catenulatum, Longum, Adolescentis	Gu et al. (Acarbose arm) [17]	↓ Glycemic, lipid profile, anthropometric results
↑: Bifidobacterium	Dentium, Pseudocatenulatum, Adolescentis, Bifidum	Wu et al. [19]	↓ Glycemic results
↑: Slackia	Heliotrinireducens, Exigua		
↑: Collinsella	Intestinalis, Aerofaciens, Stercoris		
↑: Coriobacterium	Glomerans		
↑: Corynebacterium	Jeikeium, Resistens, Pseudogenitalium, Striatum		
↑: Actinomyces	Coleocanis, oral taxon		
↑: Dermacoccus	unclassified		
↑: Propionibacterium	Acne		
↑: Rothia	Mucilaginosa		
↑: Olsenella	Uli	Murphy et al. [21]	↓ Glycemic, anthropometric results
↑: changes were present only at phylum level		Lee et al. (RYGB arm) [22]	↓ Glycemic, anthropometric results
↑: Bifidobacterium	–	Firouzi et al. [24]	↓ Glycemic results
↑: Bifidobacterium	–	Hsieh et al. [26]	↓ Glycemic, lipid profile, inflammatory results, sBP
↑: Bifidobacterium	Longum, Fragilis	Medina-Vera et al. [27]	↓ Glycemic, lipid profile, inflammatory results, FFAs
↑: Bifidobacterium	–	Pedersen et al. [28]	↓ Glycemic results
↑: Bifidobacterium	Breve	Zhang et al. (Probiotic arm) [31]	↓ Lipid profile results
↑: Bifidobacterium	Breve	Zhang et al. (Symbiotic arm) [31]	↓ Glycemic, lipid profile results

↓ – decreased abundance of genus and / or species after applied treatment. ↑ – increased abundance of genus and / or species after applied treatment. "–" means that a certain parameter was not evaluated, achieved, or provided in a specific trial. RCT – randomized controlled trial; FFAs – free fatty acid; HR – heart rate; RYGB - Roux-en-Y gastric bypass; sBP – systolic blood pressure.
