# Supplementary material for: Microbiome Changes after Type 2 Diabetes Treatment: A Systematic Review
Source: Medicina (Kaunas). 2021 Oct 11;57(10):1084. doi: 10.3390/medicina57101084 (PMC8540512; doi:10.3390/medicina57101084)
Supplement: Supplementary file 1 [file medicina-57-01084-s001.zip › medicina-1366386-supplementary/S5_table.pdf]

Table S5. Specific genera and species alterations in *Proteobacteria* phylum with corresponding clinical outcomes after any T2D treatment

Genus	Species	RCT	Achieved outcome (s)
↓: Bilophila	Wadsworthia	Gu et al. (Acarbose arm) [17]	↓ Glycemic, lipid profile, anthropometric results
↓: Enterobacter	Cloacae		
↓: Oxalobacter	Formigenes		
↓: Parasutterella	–	Tong et al. (Prebiotic arm) [18]	↓ Glycemic, lipid profile, anthropometric results
↓: Campylobacter	Gracilis, Hominis	Wu et al. [19]	↓ Glycemic results
↓: Shewanella	Frigidimarina, Putrefaciens		
↓: Haemophilus	Parainfluenzae	Zhang et al. (Prebiotic arm) [31]	↓ Glycemic, lipid profile results
↓: Haemophilus	Parainfluenzae	Zhang et al. (Symbiotic arm) [31]	↓ Glycemic, lipid profile results
↑: Escherichia/Shigella	–	Tong et al. (Metformin arm) [18]	↓ Glycemic, lipid profile, anthropometric results, ↑ dBP
↑: Klebsiella	–		
↑: Klebsiella	–	Tong et al. (Prebiotic arm) [18]	↓ Glycemic, lipid profile, anthropometric results
↑: Rheinheimera	unclassified	Wu et al. [19]	↓ Glycemic results
↑: Aeromonas	Caviae		
↑: Acinetobacter	Baumannii, Haemolyticus		
↑: Neisseria	Bacilliformis		
↑: Agrobacterium	Tumefaciens		
↑: Burkholderia	Vietnamiensis, Mallei		
↑: Citrobacter	Rodentium, Koseri, Youngae, unclassified		
↑: Pseudomonas	Syringae		
↑: Yersinia	Enterocolitica		
↑: Desulfotalea	Psychrophila		
↑: Escherichia	Coli		
↑: Salmonela	Bongori, Enterica		
↑: changes were present only at phylum level		Lee et al. [22]	↓ Glycemic, anthropometric results
↑: Escherichia	Coli	Balfego et al. [30]	↓ Glycemic, anthropometric results
↑: Bilophila	Wadsworthia	Zhang et al. (Prebiotic arm) [31]	↓ Glycemic, lipid profile results
↑: Parasutterella	Excrementihominis		
↑: Klebsiella	Oxytoca, Pneuminiae, Variicola		
↑: Enterobacter	Cloacae, Aerogenes		
↑: Citrobacter	Koseri		
↑: Escherichia	Coli		
↑: Bilophila	Wadsworthia	Zhang et al. (Symbiotic arm) [31]	↓ Glycemic, lipid profile results
↑: Parasutterella	Excrementihominis		
↑: Klebsiella	Oxytoca, Pneumoniae, Variicola group, Variicola/pneumoniae		
↑: Enterobacter	Hormaechei/cloacae, Aerogenes, Cloacae		
↑: Citrobacter	Koseri, unclassified (sp. 30 2)		
↑: Escherichia	Coli		

↓ – decreased abundance of genus and / or species after applied treatment. ↑ – increased abundance of genus and / or species after applied treatment. "–" means that a certain parameter was not evaluated, achieved, or provided in a specific trial. RCT – randomized controlled trial; dBP – diastolic blood pressure.
