# Supplementary material for: Microbiome Changes after Type 2 Diabetes Treatment: A Systematic Review
Source: Medicina (Kaunas). 2021 Oct 11;57(10):1084. doi: 10.3390/medicina57101084 (PMC8540512; doi:10.3390/medicina57101084)
Supplement: Supplementary file 1 [file medicina-57-01084-s001.zip › medicina-1366386-supplementary/S6_table.pdf]

Table S6. Specific genera and species alterations in *Verrucomicrobia*, *Euryarchaeota*, *Spirochaetes*, *Fusobacteria* phyla with corresponding clinical outcomes after any T2D treatment

**Phylum**	**Genus**	**Species**	**RCT**	**Achieved outcome (s)**
Verrucomicrobia	↑: Akkermansia	–	Cortez et al. [20]	↓ Anthropometric results
	↑: Akkermansia	–	Lee et al. [22]	↓ Glycemic, anthropometric results
	↑: Akkermansia	Muciniphila	Medina-Vera et al. [27]	↓ Glycemic, lipid profile, inflammatory results, FFAs
	↑: Akkermansia	–	Shin et al. [29]	↓ Glycemic, inflammatory results, ↑ HR
Euryarchaeota	↑: changes were present only at phylum level		Mobini et al. [23]	↓ Glycemic results
Spirochaetes	↑: Brachyspira	Pilosicoli	Wu et al. [19]	↓ Glycemic results
Fusobacteria	↓: Fusobacterium	Mortiferum	Gu et al. (Acarbose arm) [17]	↓ Glycemic, lipid profile, anthropometric results
	↓: Fusobacterium	Nucleatum (subsp. vincenti), Nucleatum (subsp. polymorphum)	Wu et al. [19]	↓ Glycemic results
	↑: Fusobacterium	–	Tong et al. (Metformin arm) [18]	↓ Glycemic, lipid profile, anthropometric results, ↑ dBP
	↑: Fusobacterium	Ulcerans, Varium	Zhang et al. (Prebiotic arm) [31]	↓ Glycemic, lipid profile results
	↑: Fusobacterium	Ulcerans	Zhang et al. (Symbiotic arm) [31]	↓ Glycemic, lipid profile results

↓ – decreased abundance of genus and / or species after applied treatment. ↑ – increased abundance of genus and / or species after applied treatment. "–" means that a certain parameter was not evaluated, achieved, or provided in a specific trial. dBP – diastolic blood pressure; FFAs – free fatty acids; HR – heart rate; RCT – randomized controlled trial.
