# Supplementary material for: Microbiome Changes after Type 2 Diabetes Treatment: A Systematic Review
Source: Medicina (Kaunas). 2021 Oct 11;57(10):1084. doi: 10.3390/medicina57101084 (PMC8540512; doi:10.3390/medicina57101084)
Supplement: Supplementary file 1 [file medicina-57-01084-s001.zip › medicina-1366386-supplementary/S8_Supplementary_material.pdf]

Supplementary material S8

Applied keywords: “type 2 diabetes mellitus”, “microbiome”, “microflora”, “microbiota”, “gut bacteria”, “probiotic”, “prebiotic”, “synbiotic”, “bariatric surgery”, “metabolic surgery”, “fecal matter”, “fecal matter transplantation”, “antidiabetic”, “drug”, “treatment”, “randomized controlled trial”.

Used search filters (whenever applicable):

- Randomized Controlled Trial.
- Publication Date: last 5 years.

Executed search strings (whenever applicable):

- (type 2 diabetes mellitus[MeSH Terms]) AND ((microbiome[MeSH Terms]) OR (microflora) OR (gut bacteria)) AND ((probiotic) OR (prebiotic) OR (synbiotic)).
- (type 2 diabetes mellitus[MeSH Terms]) AND ((microbiome[MeSH Terms]) OR (microflora) OR (gut bacteria)) AND ((bariatric surgery) OR (metabolic surgery)).
- (type 2 diabetes mellitus[MeSH Terms]) AND ((microbiome[MeSH Terms]) OR (microflora) OR (gut bacteria)) AND ((fecal matter) OR (fecal matter transplantation)).
- (type 2 diabetes mellitus[MeSH Terms]) AND ((microbiome[MeSH Terms]) OR (microflora) OR (gut bacteria)) AND ((antidiabetic) OR (drug) OR (treatment)).
- (type 2 diabetes mellitus) AND ((microbiome) OR (microflora) OR (gut bacteria)) AND ((probiotic) OR (prebiotic) OR (synbiotic)) and (randomized controlled trial).
- (type 2 diabetes mellitus) AND ((microbiome) OR (microflora) OR (gut bacteria)) AND ((bariatric surgery) OR (metabolic surgery)) AND (randomized controlled trial).
- (type 2 diabetes mellitus) AND ((microbiome) OR (microflora) OR (gut bacteria)) AND ((fecal matter) OR (fecal matter transplantation)) AND (randomized controlled trial).
- (type 2 diabetes mellitus) AND ((microbiome) OR (microflora) OR (gut bacteria)) AND ((antidiabetic) OR (drug) OR (treatment)) AND (randomized controlled trial).
